# Supplementary material for: The influence of role awareness, empathy induction and trait empathy on dictator game giving
Source: PLoS One. 2022 Mar 10;17(3):e0262196. doi: 10.1371/journal.pone.0262196 (PMC8912153; doi:10.1371/journal.pone.0262196)
Supplement: S1 Table — (DOCX) [file pone.0262196.s001.docx]

**S1 Table. Classification of answers in the imagine other task by treatment**

| Class | Empathy induction, n = 27  (word count average = 31) | Role uncertainty and Empathy induction, n = 40) (word count average = 21) |
| --- | --- | --- |
| Feelings | 25 (93 %) | 32 (80 %) |
| Negative feelings | 16 (59 %) | 18 (45 %) |
| Positive feelings | 18 (67 %) | 24 (60 %) |
| Justice/ injustice or fairness / unfairness | 14 (52 %) | 15 (38 %) |
| Focus on own choice* | 24 (89 %) | 21 (53 %) |
| Other | 0 | 1 (0.3 %) |

*Refers to focusing on the decision-maker’s own decision, not on such decisions in general
