# Supplementary material for: The influence of role awareness, empathy induction and trait empathy on dictator game giving
Source: PLoS One. 2022 Mar 10;17(3):e0262196. doi: 10.1371/journal.pone.0262196 (PMC8912153; doi:10.1371/journal.pone.0262196)
Supplement: S1 File — (DOCX) [file pone.0262196.s002.docx]

**S1 File. Trait empathy scales**

**Interpersonal Reactivity Index, IRI (Davis 1980)**

Fantasy Scale

1. When I am reading an interesting story or novel, I imagine how I would feel if the events in the story were happening to me. (FS)
2. I daydream and fantasize, with some regularity, about things that might happen to me. (FS)
3. I really get involved with the feelings of the characters in a novel. (FS)
4. After seeing a play or movie, I have felt as though I were one of the characters. (FS)
5. When I watch a good movie, I can very easily put myself in the place of a leading character. (FS)
6. I am usually objective when I watch a movie or play and I don't often get completely caught up in it. (FS) (R)
7. Becoming extremely involved in a good book or movie is somewhat rare for me. (FS) (R)

Perspective-Taking Scale

1. I sometimes find it difficult to see things from the "other guy's" point of view. (PT) (R)
2. I try to look at everybody's side of a disagreement before I make a decision. (PT)
3. If I'm sure I'm right about something, I don't waste much time listening to other people's arguments. (PT) (R)
4. I believe that there are two sides to every question and try to look at them both. (PT)
5. When I'm upset at someone, I usually try to "put myself in his shoes" for a while. (PT)
6. Before criticizing somebody, I try to imagine how I would feel if I were in their place. (PT)
7. I sometimes try to understand my friends better by imagining how things look from their perspective. (PT)

Empathic Concern Scale

1. I often have tender, concerned feelings for people less fortunate than me. (EC)
2. Sometimes I don't feel sorry for other people when they are having problems. (EC) (R)
3. When I see someone being taken advantage of, I feel kind of protective toward them. (EC)
4. Other people's misfortunes do not usually disturb me a great deal. (EC) (R)
5. I am often quite touched by things that I see happen. (EC)
6. I would describe myself as a pretty soft-hearted person. (EC)
7. When I see someone being treated unfairly, I sometimes don't feel very much pity for them. (EC) (R)

Personal Distress Scale

1. In emergency situations, I feel apprehensive and ill-at-ease. (PD)
2. I sometimes feel helpless when I am in the middle of a very emotional situation. (PD)
3. When I see someone get hurt, I tend to remain calm. (PD) (R)
4. Being in a tense emotional situation scares me. (PD)
5. I am usually pretty effective in dealing with emergencies. (PD) (R)
6. I tend to lose control during emergencies. (PD)
7. When I see someone who badly needs help in an emergency, I go to pieces. (PD)

**A Questionnaire of Cognitive and Affective Empathy, QCAE (Reniers et al 2011)**

Cognitive empathy, Perspective taking

1. I can easily work out what another person might want to talk about. 25 (CE/PT)
2. I can tell if someone is masking their true emotion. 26 (CE/PT)
3. I can sense if I am intruding, even if the other person does not tell me. 24 (CE/PT)
4. I am good at predicting how someone will feel. 19 (CE/PT)
5. I am good at predicting what someone will do. 27 (CE/PT)
6. I am quick to spot when someone in a group is feeling awkward or uncomfortable. 20 (CE/PT)
7. I can pick up quickly if someone says one thing but means another. 16 (CE/PT)
8. I can easily tell if someone else is interested or bored with what I am saying. 22 (CE/PT)
9. I can easily tell if someone else wants to enter a conversation. 15 (CE/PT)
10. Other people tell me I am good at understanding how they are feeling and what they are thinking. 21 (CE/PT)

Cognitive empathy, Online simulation

1. I try to look at everybody’s side of a disagreement before I make a decision. 3 (CE/OS), in IRI (PT)
2. Before criticizing somebody, I try to imagine how I would feel if I was in their place. 6 (CE/OS)
3. When I am upset at someone, I usually try to “put myself in his shoes” for a while. 5 (CE/OS)
4. I always try to consider the other fellow’s feelings before I do something. 30 (CE/OS)
5. I sometimes try to understand my friends better by imagining how things look from their perspective. 4 (CE/OS)
6. I can usually appreciate the other person’s viewpoint, even if I do not agree with it. 28 (CE/OS)
7. I sometimes find it difficult to see things from the “other guy’s” point of view. 1 (CE/OS) R, in IRI (PT) (R)
8. Before I do something I try to consider how my friends will react to it. 31 (CE/OS)
9. I find it easy to put myself in somebody else’s shoes. 18 (CE/OS)

Affective empathy, Emotion contagion

1. I am happy when I am with a cheerful group and sad when the others are glum. 13 (AE/EC)
2. It worries me when others are worrying and panicky. 14 (AE/EC)
3. People I am with have a strong influence on my mood. 9 (AE/EC)
4. I am inclined to get nervous when others around me seem to be nervous. 8 (AE/EC)

Affective empathy, Peripheral responsivity

1. I usually stay emotionally detached when watching a film. 29 (AE/PerR) R
2. I am usually objective when I watch a film or play, and I don’t often get completely caught up in it. 2 (AE/PerR) R
3. I often get deeply involved with the feelings of a character in a film, play, or novel. 11 (AE/PerR)
4. It is hard for me to see why some things upset people so much. 17 (AE/PerR) R

Affective empathy, Proximal responsivity

1. I often get emotionally involved with my friends’ problems. 7 (AE/ProR)
2. Friends talk to me about their problems as they say that I am very understanding. 23 (AE/ProR)
3. It affects me very much when one of my friends seems upset. 10 (AE/ProR)
4. I get very upset when I see someone cry. 12 (AE/ProR)
