# Supplementary material for: The influence of role awareness, empathy induction and trait empathy on dictator game giving
Source: PLoS One. 2022 Mar 10;17(3):e0262196. doi: 10.1371/journal.pone.0262196 (PMC8912153; doi:10.1371/journal.pone.0262196)
Supplement: S2 File — (DOCX) [file pone.0262196.s003.docx]

**S2 File. On screen instructions**

***Decision-makers***

**Baseline (role certainty, no empathy induction)**

You have been assigned into the role of a decision-maker.

You have 16 euros.

It is your job to decide, if you want to give some of the 16 euros to the recipient who has been randomly assigned to you. Should you choose to give them something, it is for you to decide how much you want to give.

Please write in the field below, how much you would like to give to the recipient.

**Role certainty and empathy induction**

You have been assigned into the role of a decision-maker.

You have 16 euros.

It is your job to decide, if you want to give some of the 16 euros to the recipient who has been randomly assigned to you. Should you choose to give them something, it is for you to decide how much you want to give.

Before you decide how much you would like to give, consider how receiving different amounts of money would make the recipient feel.

Please write here your estimation on the recipient’s feelings.

Please write in the field below, how much you would like to give to the recipient.

**Role uncertainty and no empathy induction**

You will be making your decision without knowing whether you will be assigned to the role of a decision-maker or a recipient. The participants are randomly assigned into their roles as decision-makers or recipients only after everyone has made their decision.

Make your decision thinking that you are the decision-maker, who has been given 16 euros.

It is your job to decide, if you want to give some of the 16 euros to the recipient who has been randomly assigned to you. Should you choose to give them something, it is for you to decide how much you want to give.

After all participants have made their decision, there will be a lottery. If you are chosen as the decision-maker, your randomly selected recipient will get the amount you gave them, and you get to keep the sum you left to the decision-maker. If you are randomly chosen to be a recipient, however, you will get the sum that your randomly assigned decision-maker has chosen to give to the recipient.

Please write in the field below, how much you would like to give to the recipient.

**Role uncertainty and empathy induction**

You will be making your decision without knowing whether you will be assigned to the role of a decision-maker or a recipient. The participants are randomly assigned into their roles as decision-makers or recipients only after everyone has made their decision.

Make your decision thinking that you are the decision-maker, who has been given 16 euros.

It is your job to decide, if you want to give some of the 16 euros to the recipient who has been randomly assigned to you. Should you choose to give them something, it is for you to decide how much you want to to give.

After all participants have made their decision, there will be a lottery. If you are chosen as the decision-maker, your randomly selected recipient will get the amount you gave them, and you get to keep the sum you left to the decision-maker. If you are randomly chosen to be a recipient, however, you will get the sum that your randomly assigned decision-maker has chosen to give to the recipient.

Before you decide how much you would like to give, consider how receiving different amounts of money would make the recipient feel.

Please write here your estimation on the recipient’s feelings.

Please write in the field below, how much you would like to give to the recipient.

***Recipients***

You have randomly been assigned to the role of the recipient. You will not make decision in this experiment. The sum your randomly assigned decision-maker gives you will soon be shown on the screen.
