# Supplementary material for: The influence of role awareness, empathy induction and trait empathy on dictator game giving
Source: PLoS One. 2022 Mar 10;17(3):e0262196. doi: 10.1371/journal.pone.0262196 (PMC8912153; doi:10.1371/journal.pone.0262196)
Supplement: Attachment — Submitted filename: Response to reviewers.docx [file pone.0262196.s005.docx]

**Responses to review comments, 2^nd^ round**

The influence of role awareness, empathy induction and trait empathy on dictator game giving

**Comments and responses**

**Comment:** Reviewer #1: Thanks for updating and revising many of the points raised in the prior review. As stated previously, the authors have explored an interesting research question spanning many fields including empathy, prosocial behavior, altruism, and decision-making.

The authors have for the most part done well to address many of the points raised in the previous review. I find their new additions to significantly improve the readability of the manuscript. However, small areas of clarification remain, mainly in relation to clarity of the research motivation, discussion, and statistical analyses. Many of these were comments regarding clarification I had raised in the previous review, which I still feel the authors can improve. Note that my review is fairly specific in nature, as I do not read any major or new concerns with the paper as it stands. Hence, I detail below a few more minor revisions for the authors before publication:

**Comment:** Please add an interpretation sentence of the results at the end of the abstract.

**Response:** An interpretation sentence in the end of the abstract is now added.

**Comment:** The motivation/hypotheses still read a bit disorganized. One helpful approach I would recommend is to shorten the text in the introduction (~8 pages). This is what I had meant by streamlining rather than just pasting in new text. Clarification would also be helpful. For example, in lines 101 – 106, the authors state “positions in the income distribution are randomly decided after choices are made.” Please elaborate on “positions” (recipient positions?) and make this more accessible to the layreader. It may seem minor, but is a premise and motivation behind your manipulation of the first factor.

**Response:** We shortened the introduction (what comes before hypotheses) and clarified expressions.

**Comment:** Results: Please provide a sample size justification.

**Response:** The sample size is now justified in the text.

**Comment:** In your statistical reports of the analyses, please consider a better name for the role uncertainty factor. I say this because you term one of your levels of the factor, “role uncertainty”, which can get quite confusing for the reader. Maybe consider using “role type” as the name for the factor, and “role certainty” and “role uncertainty” as the two levels (e.g., line 380).

**Response:** Thank you for this comment. You are absolutely right. “Role type” might be a bit problematic also because some readers might confuse it with the dictator vs receiver roles. Therefore, we ended up naming this factor as “role awareness”. Related to this issue we also renamed the four conditions. In the previous version of the manuscript, we called these *Baseline*, *Role Uncertainty*, *Empathy Induction*, and *Empathy Induction plus Role Uncertainty*. In the present version of the ms, we simply use the level combinations of the two main factors in the names. Thus, they are: Role certainty/No empathy induction, Role certainty/Empathy induction, Role uncertainty/No empathy induction, and Role uncertainty/Empathy induction (abbreviations: RC/NEI, RC/EI, RU/NEI, and RU/EI). We have also changed the title of the ms accordingly, and it now has role awareness instead of role uncertainty.

**Comment:** Lines 379 – 382. I do not follow here. The authors state: The results revealed that the main effect of the role uncertainty treatment was highly significant, F(1, 127)= 17.44, p=0.000055. In other words, role uncertainty increased giving over and above the increase that empathy induction caused”

- Why do the authors not report the direction of this main effect, and instead take a regression-like interpretation of the ANOVA?

**Response:** We have now changed the way the results are reported to more closely match that of a standard ANOVA reporting. Additionally, we present the descriptive report and compare the proportions of ‘altruistic’ dictators between treatment conditions (those sending at least half of their initial allocation) only after the ANOVA results.

**Comment:** Minor: I would recommend abbreviating RU and EI at the beginning of the results, and not in the middle (i.e., line 392)?

**Response:** RU and EI are now abbreviated at the beginning of the results. The respective abbreviations of the levels of the manipulated variables (role certainty, role uncertainty or RC/RU and empathy induction, no empathy induction or EI/NEI) are now consistently used throughout the manuscript, including all the tables.

**Comment:** Starting at line 576, if both empathy induction and role uncertainty can be interpreted as methods for perspective-taking as the authors claim, then a discussion of why empathy induction was not significant here is warranted. I would suggest merging text from the paragraph starting at 586 with this paragraph. They could make the claim of inducing more cognitive-like aspects from empathy induction or the ‘justification’ of behavior aspect driving the lack of significance in the empathy induction manipulation. This would make the discussion also read less disjointed.

**Response:** We merged the last two paragraphs, and somewhat edited the paragraph.

**Comment:** It would be helpful if the authors copy and remove some of the text from the paragraph in the discussion section starting at line 490, which would be a great starting point for the paragraph with the effect size test in the results

**Response:** We removed some text about statistical power from discussion, it is now right above the results section, and we also shortned the paragraph on statistical power in discussion.
